# Prevalence and correlates of internalizing and externalizing mental health problems among in-school adolescents in eastern Ethiopia: a cross-sectional study

**DOI:** 10.1038/s41598-024-54145-2

**Published:** 2024-02-12

**Authors:** Gari Hunduma, Yadeta Dessie, Biftu Geda, Tesfaye Assebe Yadeta, Negussie Deyessa

**Affiliations:** 1 School of Nursing and Midwifery, College of Health and Medical Sciences, Haramaya University, Harar, Ethiopia; 2 School of Public Health, College of Health and Medical Sciences, Haramaya University, Harar, Ethiopia; 3 School of Nursing and Midwifery, College of Health and Medical Sciences, Madda Walabu University, Shashamene, Ethiopia; 4 Department of Preventive Medicine, School of Public Health, College of Health Sciences, Addis Ababa University, Addis Ababa, Ethiopia

**Keywords:** Mental health problems, Internalizing, Externalizing, In-school adolescents, Prevalence, Correlates, Ethiopia, Health care, Medical research, Risk factors

## Abstract

Adolescent’s mental health issues are a major social burden and a significant public health issue, but they have not received enough attention in Ethiopia. Therefore, this study aimed to determine the prevalence and correlates of internalizing and externalizing mental health problems among in-school adolescents in the Harari region, eastern Ethiopia. A cross-sectional study was conducted among 3227 in-school adolescents. Multistage sampling was used to select schools and eligible students to participate in the study. A guided, self-administered strength and difficulty questionnaire measured mental health problems. Data were double-entered, validated, and cleaned using EpiData 3.1 and analyzed using STATA version 17. Ordinal logistic regression analysis was performed to estimate the adjusted odds ratio between mental health problems and their correlates. Statistical significance was set at p-value < 0.05. The magnitudes of mental health problems among in-school adolescents by subscale was 24.17% (95% CI 22.72; 25.67) for internalizing and 11.93% (95% CI 10.85; 13.09) for externalizing problems. A high internalizing problem score was associated with females, rural residents, alcohol users, attending public schools, those bullied at school, and those in the lowest wealth index. Likewise, the likelihood of a high externalizing problem score was high among alcohol users, adolescents whose fathers are uneducated, rural, and bullied at school. The study suggests that mental health problems are prevalent among in-school adolescents in Ethiopia, especially internalizing problems. The study also identifies several risk factors associated with internalizing and externalizing problems, such as wealth index, school types, alcohol use, bullying, and rural residence. These factors may indicate the need for more mental health awareness and support programs for adolescents in Ethiopia. This highlights that schools and communities should prioritize mental health awareness and support programs for adolescents. These programs should be tailored to address the specific needs of the population, such as rural residents, those in the lowest wealth index, and those who have experienced bullying.

## Introduction

Adolescent mental health problems are often operationalized into two empirically derived constructs: externalizing and internalizing problems^1^. Internalizing problems, primarily composed of depression and anxiety, are characterized by inward-facing symptoms that affect an individual’s internal emotional state^2,3^. On the other hand, externalizing problems, which include conduct problems, hypersensitivity, inattentiveness, impulsivity, and disruptive disorders, are characterized by outward behaviors that affect an individual’s social environment^2,3^.

Adolescence is a critical period of physical, psychological, social, and cognitive development^4^. This developmental period, characterized by role confusion, high levels of stress, and emotional instability, can exacerbate these internalizing and externalizing problems, leading adolescents to engage in risky behaviors and experience poor mental health^5^.

The most common mental health problems among adolescents include conduct disorders (CD), hyperactivity/inattention difficulties, depressive disorders, and anxiety disorders^6,7^. These problems frequently coexist, and their symptoms are strikingly similar^8,9^. Furthermore, these symptoms can persist into young adulthood through homotypic or heterotypic continuity^10^, increasing the likelihood of adult mental illnesses^11–13^.

Depression, anxiety, and suicide affect 10–20% of children and adolescents worldwide, accounting for 16% of the total adolescent disease burden. As indicated in the global report of 2019, more than 13% of 1.2 billion adolescents had mental disorders^14,15^. One in every seven children and adolescents (14.3%) in Sub-Saharan Africa (SSA) suffers from major psychological problems, with nearly 10% qualifying for a psychiatric diagnosis^16^. Likewise, it ranged from 12 to 25% among children and adolescents in Ethiopia^17^. Adolescent mental health issues have become increasingly important over the last few decades^18,19^. This is also highlighted in sustainable development goal (SDG) Target 3.4 which calls for reducing noncommunicable diseases (NCD) related to premature mortality by one-third by 2030 through mental health promotion, prevention, and treatment. Furthermore, there is growing support for placing mental health at the forefront of development and health agendas^20^ and there are global initiatives to raise the profile of mental health^21–23^.

Adolescents with poor mental health conditions may experience a heightened sensitivity to social rejection, stigma, educational difficulties, physical and psychological ill-health, risk-taking behaviors, human rights violations, and limiting opportunities to lead fulfilling lives as adults^24^. Most high-risk behaviors such as self-harm, substance use, and risky sexual behavior occur due to mental health problems^25,26^. Domestic violence and delinquent behaviors that may persist throughout the life course are also the results of poor adolescent mental health^25,26^.

Evidence revealed that socio-demographic (age, sex, residence, religion; parental education, parental occupation, family income)^27–29^, risky behaviors (substance use, suicidal behavior)^30–32^, biological (chronic physical illness and family history of mental illness)^33,34^, and psychosocial (family structures, family loss, marital discordance, violence, bullying, low self-esteem, adverse and childhood maltreatment)^35–42^ were factors associated with both internalizing and externalizing mental health problems among adolescents.

The global health crisis, COVID-19, has had far-reaching effects beyond the immediate threat to physical health. It has significantly altered the daily routines, social interactions, and educational experiences of adolescents. This demographic, already in a critical stage of psychological development, has been particularly affected. According to a scientific brief released by the World Health Organization (WHO), the global prevalence of anxiety and depression increased by 25% in the first year of the COVID-19 pandemic^43^. studies conducted in the United States showed that COVID-19 had worsened the psychological and behavioral problems of adolescents compared to the time before the pandemic^44,45^. Studies conducted in Italy and Spain also reported increased mental health problems like conduct problems, irritability, and loneliness during the COVID-19 lockdown^46,47^. Furthermore, studies from China^48,49^, India^50,51^, and Brazil^52^ reported that mental health problems among adolescents were increasing during the COVID-19 pandemic and they underline that COVID-19 had negative impacts on adolescent mental health. Another study also found that many adolescents are often worried about the COVID-19 pandemic and its consequences^53^. While this study primarily focuses on the prevalence and correlates of internalizing and externalizing mental health problems among in-school adolescents, it is important to acknowledge the potential exacerbating effects of the pandemic on these issues.

Although the burden, consequences, and contributing factors of adolescent mental health problems are well-studied in high-income countries, data in low and middle-income countries, including Ethiopia, are insufficient. The few studies in Ethiopia focused on mixed age groups^54–56^ rather than examining adolescents aged 10–19 years. As a result, this study aimed to identify the prevalence of internalizing and externalizing mental health problems and related factors among in-school adolescents in the Harari region of Eastern Ethiopia.

Adolescent’s mental health is also highly overlooked during planning and implementation strategies. For instance, a National Strategic Plan for Adolescent and Youth Health developed in 2016 calls for reducing suicide and depression among adolescents and youth by half by 2020^17^, but the target was not achieved and no evidence was available in the Harari region^57^. Therefore, the findings from these adolescent-focused studies are critical for informing the design and implementation of appropriate policies and programs, including resource allocation, to improve adolescent mental health prevention and promotion.

According to a study conducted in the Harari region, approximately one in four adolescents had a low health-related quality of life^58^ that was significantly impacted by internalizing and externalizing mental health problems^58^. This highlights the need for further research to identify the correlates of these problems in the region and develop effective interventions to improve adolescents’ overall quality of life. Moreover, the National Strategic Plan for Adolescent and Youth Health developed in 2016 calls for reducing suicide and depression among adolescents and youth by half by 2020, but the target was not achieved or no evidence was available in the region^58^. Therefore, this study helps to identify the factors associated with mental health problems and develop effective interventions to address them, which can have a positive impact on adolescent mental health across Ethiopia. It also contributes to achieving the national adolescent and youth health strategy targets for 2020–2025 by 2025^59^.

## Method and materials

### Study setting and design

A school-based cross-sectional study was conducted in the Harari region located in Eastern Ethiopia at 511 km from Addis Ababa. Unlike most other regions in Ethiopia, the majority (54.2%) of people in the region live in urban areas^60^. The region’s capital is the ancient ‘city of Harar’, a predominantly Muslim city where people specialize in trading. It is also believed to be the holiest city for Islam due to its rich collection of important Islamic monuments notably including 82 mosques and 102 shrines^61^. Trade is the main source of revenue for people in the region. Psychoactive substances like *khat* (Catha edulis), tobacco, and coffee constitute a substantial share of trading activities. Linked to this, there is a high prevalence of khat chewing in the community where about a quarter of young people do it^60^. Most of the rural population of the region depends on rain-fed and small-scale farming. Khat is the dominant cash crop in most of the rural sub-districts of Harar^61–63^. During the study period, there were 112 schools in the region, of which 85, the target population for our study, have both primary (7th through 8th grades) and secondary (9th through 12th grades) level students^64^. Twenty-three schools, from urban and rural, both public and private, were included in this study. The data was collected from November 24 to December 31, 2020.

### Population and sampling

The source population included all in-school adolescents in Harari Region, whereas adolescents in the randomly selected schools during the study period constituted the study population. The sample size was calculated using OpenEpi stat software with the assumption that the average prevalence of childhood mental health problems in Ethiopia is (18.5%)^65^, degree of precision (d) = 2%, 95% Confidence level, design effect of 2 and 15% non-response rate, the sample became 3326.

From the total of 83, 23 schools from both urban and rural areas, both public and private, were selected to be involved in this study. A multi-stage sampling technique using simple random sampling was used to select schools and study participants. First, the schools were stratified into urban versus rural, public schools versus private schools, and primary versus secondary schools. Then, 23 schools representing all the strata were randomly selected using the lottery method proportionally. Finally, from each grade level of each school, sections were randomly chosen by lottery method considering the number of sections and students in those sections and all students of the selected sections were included in the study.

### Data collection

A structured and standardized guided self-administered questionnaire was used for collecting data. The questionnaires were initially developed in English and translated into Amharic and Afan Oromo, the region’s two most widely spoken languages. The backward and forward translation technique was utilized to maintain uniformity throughout translations. The final version was reviewed by mental health professionals and English, Amharic, and Afan Oromo language experts. The questionnaires were also reviewed by a panel of experts familiar with the local culture and context to assess the relevance and appropriateness of each item in the Ethiopian context. The questionnaire was pre-tested, and Cronbach's alpha for reliability and validity was checked before the primary data collection. The report showed an acceptable (> 70%) reliability coefficient.

The data were collected from adolescent students at their schools. An appropriate setting (rooms) was facilitated for students if their section was not covenant to fill out the questionnaire. Fortunately, the maximum class size for the year is 25 students because of the COVID-19 pandemic class arrangement, so a small number of students (not more than 25) were assigned to participate in one session. Orientation was given to participants about the study and how to fill out the questionnaire to maintain the data quality. Two data collectors were assigned per session to facilitate and guide the respondents as appropriate.

### Variables and measurements

#### Dependent variables

##### Mental health problems

Internalizing (emotional symptoms and peer relationship problems) and externalizing (conduct problems, hyperactivity, or inattention) problems are measured using the strength and difficulty questionnaire (SDQ-25)^66,67^. SDQ has 25 items categorized into 5 different subscales: 4 subscales contributing to a total difficulties score and the fifth subscale to identify strengths. Each item has three response options: “Not True,” “Somewhat True,” and “Certainly True”. Each item is answered on 3- a Likert scale ranging from ‘Not true’ (rated as 0), ‘Somewhat true’ (rated 1) to ‘Certainly true’ (rated 2). ‘Somewhat true’ is always noted as 1, but the notation of ‘Not true’ and ‘Certainly true’ varies depending on the scale elements^68^.

The scoring was performed using the predictive algorithm converted into STATA syntax available on the SDQinfo website^69,70^. Higher scores on the SDQ scale mean a greater risk of mental health problems. By applying the method of score banding reported by Goodman, the self-completed version of the SDQ total difficulties score was categorized into ‘Normal’ (0–15), ‘Borderline’ (16–19), and ‘Abnormal’ (20–40) scores^37,68^. The banding for each subcategory was described as follows. For emotional problems, the categories are Normal (0–5), Borderline (6), and Abnormal (7–10) scores. For conduct problems, the cut-off points for the Normal, Borderline, and Abnormal categories were (0–3), (4), and (5–10), respectively. The hyperactivity problems subscale category is Normal (0–5), Borderline (6), and Abnormal (7–10), while the peer problem subscale for Normal, Borderline, and Abnormal are (0–3), (4–5), and (6–10), respectively. For prosocial behaviors, the cut-off point for the Normal, Borderline, and Abnormal categories were (6–10), (5) and (0–4) respectively^68^.

For each of the five subscales, the score ranged from 0 to 10. The sum of the first four problem domains (excluding the prosocial behavior items) was used to generate total difficulties score ranging from 0 to 40, which is further categorized as Normal (score ≤ 15), Borderline (score 16–19), and Abnormal (score 20–40). The total SDQ was used and it showed a Cronbach’s α of 0.73. The alpha coefficients for each problem subscale were 0.53 for emotional symptoms, 0.51 for conduct problems, 0.46 for hyperactivity, and 0.31 for peer problems subscale.

The sum of conduct and hyperactivity scales was used to generate an externalizing score ranging from 0 to 20 and the sum of emotional and peer problem scales to generate an internalizing score ranging from 0 to 20. The internalizing problem subscale category is normal (0–7), borderline (8), and abnormal (9–20) while the externalizing subscale for normal, borderline, and abnormal is (0–8), (9), (10–20), respectively^71,72^. A borderline category score was considered a cut-off point for each difficulty sub-score for indicating mental health problems in this study. The Cronbach’s α for SDQ is 0.73 in the current sample.

#### Independent variables

Socio-demographic, psychosocial, behavioral, and biological correlates data were assessed using a standard questionnaire derived from previous literature. Socioeconomic status including parents’ educational level, occupation, and wealth, was also considered and indexed. The wealth index was measured using the number and specific goods (such as televisions, bicycles, and cars; materials used for housing construction such as flooring material; type of drinking water source; and toilet and sanitation facilities) owned by the family as reported by the adolescents; this data was analyzed using the principal component analysis^73^.

Substance use: It is defined as the ever-current use of substances like alcohol drinking, cigarette smoking, khat chewing, or other illicit drugs. We used an adapted questionnaire from previous studies from the Global School-Based Health Survey (GSHS) to assess their use status, frequency, and when they started using substances. This scale, which consists of nine items, measures adolescent students’ use of substances and the frequency of their use in the past month.

Family history of mental disorders: we asked participants if they had first- and/or second-degree relatives who had experienced mental health problems.

The family-related psychosocial variables, including living arrangements, manner of upbringing, and parent’s living situation, were assessed using a standard questionnaire based on the literature.

### Data quality control

For all data collectors and supervisors, the training was undertaken for 5 days regarding collecting the data. All data collection tools were pre-tested at Dire Dawa among 5% of the sample size with similar school adolescent students. Appropriate modifications were made to make them consistent and clear before using them for the actual data collection. The data collection process was closely supervised daily by trained supervisors and principal investigators. Data editors were assigned to check for missing and inconsistencies for further cleaning before entry. Finally, the completed data were double entered by different data entry clerks for validation and reduction of error due to entering.

### Data analysis

The data were double-entered, validated, and cleaned using EpiData 3.1 and analyzed using STATA 14.1. Descriptive statistics, including mean, standard deviations, and percentages, were performed to characterize the sample regarding socio-demographic characteristics and outcome variables. Chi-squared tests were used to determine the association between the proportions of mental health problems among different groups of study participants. Further, multivariable ordinal logistic regression (OLR) was performed to estimate the adjusted odds ratio (AOR) between socio-demographic data and mental health problems, including emotional, conduct, peer, and hyperactivity problems, and exhibiting prosocial behaviors. The ordinal levels were normal, borderline, and abnormal. The parallel lines assumption and brant supported the OLR model. Independent variables were age, sex, residence, religion, school type, marital status, family size, family structure, parental education, and parental occupations. Statistical significance was declared at (P < 0.05).

### Ethical considerations

Ethical approval was obtained from the Institutional Health Research Ethics Review Committee (IHRERC) of Haramaya University, College of Health and Medical Sciences with Ref. No IHRERC/149/2019. Respondents, parents/guardians, and school administrators were given complete and accurate information about the study, including the purpose, procedures, risks, and benefits. In addition, the adolescents were informed their participation in the study was entirely voluntary, that not participating would have no negative consequences for their family or the adolescents, and that they could stop at any point or skip questions they did not want to answer.

For participants aged 13 to 17, we obtained written voluntary assent from the adolescents and a written informed and signed voluntary consent from one of their parents or guardians. Those participants who were 18 years and older provided their consent. Personal identifiers were not included in the written questionnaires to ensure participants’ confidentiality. All data collected were anonymized and kept on a personal computer protected with a password. All information remained anonymous and confidential. Both participants and their parents were informed that the information gathered would be disseminated to assist in knowledge generation only. The study was conducted following the Declaration of Helsinki—Ethical principles for medical research involving human subjects.

## Results

### Socio-demographic characteristics of participants

A total of 3227 in-school adolescents were included in this study giving a 97% response rate. The mean age of the respondents was 15.69 (SD ± 1.79) years. The majority, 2706 (83.85%) of the respondents were from urban areas and 2302 (71.34%) lived with both biological parents. More than half, 1670 (51.75%) were girls, 1540 (50.82%) were from primary schools, and 1749 (54.2%) were Muslims. More than two-thirds, 2162 (67%) were from public schools. A total of 1622 (50.27%) of the mothers and 1488 (46.12%) of the fathers of participants did not attend formal education; 1307 (40.5%) of the fathers and 773 (23.96%) of the mothers of participants employed. The same number, 921 (28%) of the fathers and mothers of participants were merchants; 556 (17.23%) fathers, and 1018 (31.55%) mothers participants were farmers and housewives, respectively (Table 1).

**Table 1:** Socio-demographic, characteristics of the respondents (N = 3227), in Harari regional state, Eastern Ethiopia, 2020.

Variables	Categories	Frequency	Percent
Sex	Male	1557	48.30
	Female	1670	51.70
Age	≤ 13 years	472	14.63
	14–16 years	1579	48.93
	17–19 years	1176	36.44
Residence	Urban	2706	83.85
	Rural	521	16.15
Living with	Both parents	2302	71.34
	One parent	529	16.39
	Others	396	12.27
Marital status	Never married	2948	91.35
	Married or living together	194	6.01
	Divorced/separated/widowed	85	2.63
Religions	Muslim	1749	54.20
	Cristian	1478	45.80
School type	Public	2162	67.00
	Private	1065	33.00
Family size	Below six	1651	51.16
	Six and above	1576	48.84
Father educational status	No education	1488	46.11
	Primary	331	10.26
	Secondary	489	15.15
	More than secondary	919	28.48
Mother educational status	No education	1622	50.26
	Primary	434	13.45
	Secondary	492	15.25
	More than secondary	679	21.04
Father occupation	Employed	1307	40.50
	Merchants	929	28.79
	Agricultures	556	17.23
	Others daily labors	435	13.48
Mother occupation	Employed	773	23.95
	Housewife	1018	31.55
	Merchants	921	28.54
	Others daily labors	515	15.96
Parental marital status	Living together	2453	76.01
	Living separated	240	7.44
	Divorced/separated/widowed	534	16.55

### Prevalence of adolescent’s mental health problems

**Figure 1:**
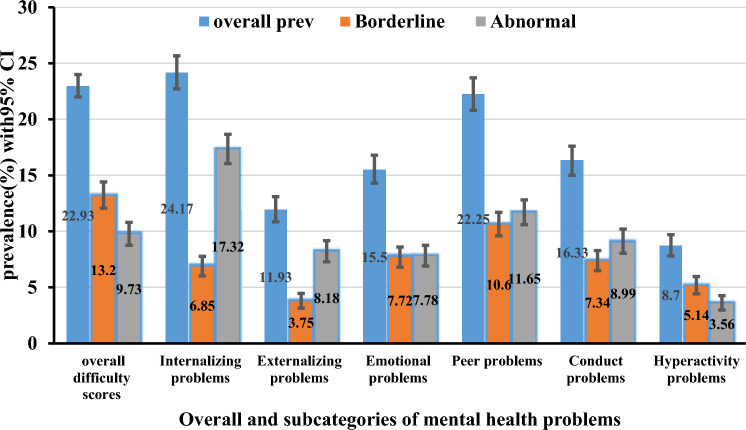
Magnitude of the overall and sub-categories of mental health problems among in-school adolescents (N = 3227), in Harari region, eastern Ethiopia, 2020.

**Figure 2:**
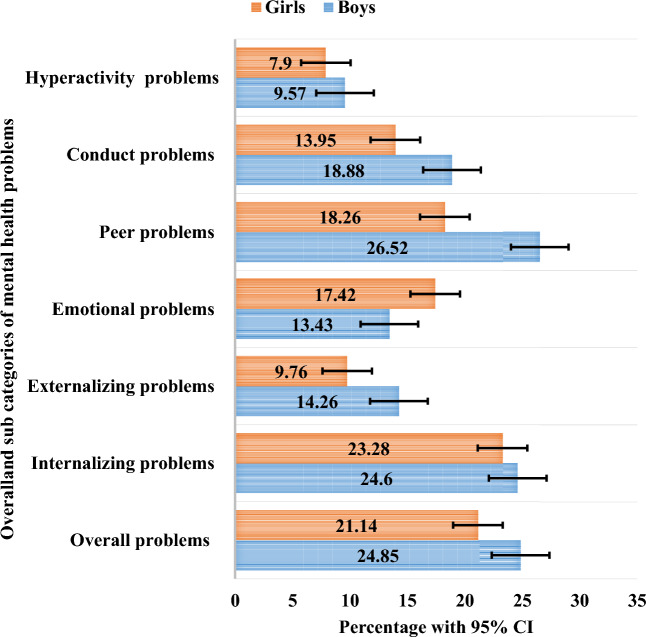
Magnitude of in-school adolescent boys and girls with mental health problems (N = 3227), in Harari region eastern Ethiopia, 2020.

Among the total of 3227 in-school adolescents, 740 (22.93%) participants had a high SDQ score, indicating an increased risk of mental health problems. The SDQ cut-off points for adolescents showed that 426 (13.20%) of the respondents scored between 16 and 19 (borderline), and 314 (9.73%) scored above 20 (abnormal), with a maximum score of 32. According to the subscale, the magnitude of internalizing problems was 24.17% (95% CI 22.72; 25.67) and externalizing problems was 11.93% (95% CI 10.85; 13.09). We conducted further analysis for each SDQ subscale to describe the proportion of students considered borderline and abnormal (Fig. 1). In addition, adolescent boys have a higher proportion of mental problems than girls in all problem categories except emotional problems (Fig. 2).

### Association between independent correlates and mental health problems

A high level of internalizing problem score was more likely among being female (AOR = 1.24, 95% CI 1.05; 1.47), rural residents (AOR = 1.74, 95% CI 1.37; 2.21), alcohol users (AOR = 1.46, 95% CI 1.10; 1.95), attending public schools (AOR = 1.27, 95% CI 1.03; 1.56), being bullied at school (AOR = 1.65, 95% CI 1.29; 2.10) and being in the lowest wealth index (AOR = 1.33, 95% CI 1.04; 1.72) (Table 2). Likewise, the likelihood of a high-level externalizing problem score was high among alcohol users (AOR = 1.82, 95% CI 1.27; 2.60); adolescents whose fathers’ are not educated (AOR = 1.71, 95% CI 1.16; 2.49); being rural (AOR = 2.16, 95% CI 1.61; 2.92) and being bullied at school (AOR = 1.79, 95% CI 1.32; 2.43) (Table 3).

**Table 2:** Ordinal logistic regression model showing the correlates of internalizing problems among in-school adolescents (N = 325) in Harari Region, Eastern Ethiopia, 2020.

Variables	Internalizing problem status			Crude analysis	Model I	Model II	Model III	Model IV
	Normal (%)	Borderline (%)	Abnormal (%)	COR (95% CI)	AOR (95% CI)	AOR (95% CI)	AOR (95% CI)	AOR (95% CI)
Sex								
Male	75.40	7.32	17.28	1	1			1
Female	76.23	6.41	17.37	0.97 (0.82; 1.13)	1.07 (0.92; 1.27)			**1.24 (1.05; 1.47)**
Age								
≤ 13 years	74.58	6.14	19.28	1	1			1
14–16 years	78.66	6.14	15.20	**0.79 (0.62; 0.99)**	0.82 (0.64; 1.05)			0.85 (0.66; 1.10 )
17–19 years	72.53	8.08	19.39	1.10 (0.85; 1.39)	1.11 (0.86; 1.43)			1.07 (0.83; 1.39)
Marital status								
Single	76.80	6.82	16.38	1	1			1
Married	65.59	7.17	27.24	**1.78 (1.38; 2.31)**	**1.59 (1.22; 2.07)**			1.32 (0.99; 1.74)
Alcohol drinking								
Never drink	77.23	6.70	16.06	1	1			1
Ever drink	64.74	7.99	27.27	**1.87 (1.49; 2.36)**	**1.36 (1.03; 1.82)**			**1.46 (1.10; 1.95)**
Tobacco use								
Never use	76.99	6.54	16.47	1	1			1
Ever use	56.76	11.89	31.35	**2.46 (1.83; 3.31)**	**1.52 (1.04; 2.23)**			0.87 (0.58; 1.31)
Khat use								
Never use	77.36	6.27	16.37	1	1			1
Ever use	67.77	9.90	22.33	**1.59 (1.29; 1.94)**	1.16 (0.92; 1.48)			1.02 (0.79; 1.30)
Social media use status								
None hours	74.86	7.52	17.62	1	1			1
1 to < 3 h	41.72	34.39	30.05	**0.71 (0.59; 0.86)**	**1.46 (1.20; 1.76)**			**0.74 (.61; 0.91)**
3 to < 5 h	7.19	8.14	8.23	1.10 (0.79; 1.48)	**1.41 (1.02; 1.94)**			0.89 (.62; 1.24)
5 h and above	13.24	15.38	22.72	**1.52 (1.22; 1.91)**	**1.94 (1.53; 2.46)**			1.15 (.89; 1.48)
Father’s educational status								
No education	71.98	7.12	20.90	**1.70 (1.39; 2.08)**		**1.37 (1.05; 1.79)**		1.13 (0.86; 1.50)
Primary	72.81	9.37	17.82	**1.58 (1.18; 2.12)**		1.26 (0.91; 1.75)		1.10 (0.78; 1.55)
Secondary	79.35	6.34	14.31	1.13 (0.85; 1.48)		1.03 (0.77; 1.39)		1.01 (0.74; 1.36)
Above secondary	81.28	5.77	12.95	1		1		**1**
Mother’s educational status								
No education	74.19	8.53	17.28	**1.48 (1.19; 1.85)**		0.98 (0.74; 1.30)		0.95 (0.72; 1.27)
Primary	80.08	5.28	14.63	**1.38 (1.04; 1.83)**		1.12 (0.81; 1.54)		1.08 (0.78; 1.51)
Secondary	79.97	6.92	13.11	1.01 (0.75; 1.35)		1.00 (0.74; 1.36)		1.01 (0.73; 1.34)
Above secondary	74.19	8.53	17.28	1		1		**1**
Family size								
One to three	79.19	5.74	15.07	1		1		**1**
Four to seven	76.95	6.54	16.51	1.13 (0.88; 1.46)		1.21 (0.93; 1.58)		1.16 (0.88; 1.52)
Eight and above	69.98	8.58	21.44	**1.61 (1.21; 2.14)**		**1.47 (1.10; 2.00)**		1.23 (0.91; 1.69)
Household wealth								
Lowest	68.86	8.83	22.31	**1.917 (1.52; 2.41)**		**1.61(1.26; 2.04)**		**1.33 (1.04; 1.72)**
Middle	80.15	5.71	14.13	1.10 (0.83; 1.34)		1.05 (0.83; 1.33)		1.03 (0.81; 1.32)
Highest	81.12	5.15	13.73	1		1		**1**
Currently living with								
Both parents	76.46	7.12	16.42	1		1		**1**
One parent	74.48	6.05	19.47	1.13 (0.91; 1.40)		1.16 (0.93; 1.46)		1.19 (0.94; 1.50)
Others	73.99	6.31	19.70	1.16 (0.91; 1.48)		1.17 (0.91; 1.50)		1.12 (0.85; 1.43)
Presence of mental illness in the family								
No	78.11	6.43	15.46	1		1		**1**
Yes	60.29	9.69	30.02	**2.35 (1.89; 2.90)**		**2.10 (1.68; 2.60)**		**1.51 (1.20; 1.92)**
Residence								
Urban	79.19	6.21	14.60	1			1	1
Rural	58.35	10.17	31.48	**2.71 (2.23; 3.28)**			**2.14 (1.75; 2.63)**	**1.74 (1.37; 2.21)**
School type								
Private	72.43	7.49	20.07	1			1	1
Governmental	82.72	5.54	11.74	**1.83 (1.52; 2.20)**			**1.43 (1.17; 1,72)**	**1.27 (1.03; 1.56)**
Frequency of being bullied by others								
Not bullied	79.21	6.04	14.75	1			1	1
Once per-week	60.43	10.79	28.78	**2.44 (1.73; 3.45)**			**2.01 (1.42; 2.86)**	**1.57 (1.08; 2.29)**
Twice or more per week	61.57	10.19	28.24	**2.35 (1.92; 2.88)**			**2.03 (1.64; 2.51)**	**1.65 (1.29; 2.10)**

Bold numbers indicate statistically significant (p-value < 0.05) association.

**Table 3:** Ordinal logistic regression model showing the correlates of externalizing problems among in-school adolescents (N = 325) in Harari Region, Eastern Ethiopia, 2020.

Variables	Externalizing problem status			Crude analysis	Model I	Model II	Model III	Model IV
	Normal (%)	Borderline (%)	Abnormal (%)	COR (95% CI)	AOR (95% CI)	AOR (95% CI)	AOR (95% CI)	AOR (95% CI)
Sex								
Male	85.74	4.50	9.76	**1.53 (1.24; 1.90)**	1			1
Female	90.24	3.05	6.71	1	**1.39 (1.11; 1.74)**			1.19 (0.94; 1.51)
Age								
≤ 13 years	85.59	4.66	9.75	1	1			1
14–16 years	88.79	3.42	7.79	0.75 (0.55; 1.02)	0.79 (0.58; 1.08)			0.93 (0.67; 1.28)
17–19 years	88.10	3.83	8.08	0.80 (0.58; 1.09)	0.75 (0.54; 1.04)			0.83 (0.59; 1.16)
Marital status								
Single	88.74	3.70	7.56	**1**	1			1
Married	81.00	4.30	14.70	**1.88 (1.36; 2.58)**	**1.60 (1.15; 2.22)**			1.29 (0.91; 1.84)
Alcohol drinking								
Never drink	89.53	3.35	7.12	1	1			1
Ever drink	76.58	6.89	16.53	**2.60 (1.99; 3.41)**	**1.75 (1.24; 2.47)**			**1.82 (1.27; 2.60)**
Tobacco use								
Never use	89.15	3.48	7.36	1	1			1
Ever use	70.27	8.11	21.62	**3.47 (2.49; 4.84)**	**1.75 (1.11; 2.76)**			0.94 (0.57; 1.54)
Khat use								
Never use	89.12	3.61	7.26	1	1			1
Ever use	82.52	4.47	13.01	**1.75 (1.35; 2.26)**	1.10 (0.79; 1.51)			0.93 (0.66; 1.30)
Social media use status								
None hours	88.68	3.96	7.36	1	1			1
1 to < 3 h	90.04	2.92	7.04	0.87 (0.67; 1.13)	0.84 (0.65; 1.09)			0.85 (0.64; 1.11)
3 to < 5 h	88.75	2.08	9.17	1.01 (0.65; 1.57)	0.91 (0.58; 1.42)			0.75 (0.46; 1.20)
5 h and above	81.03	6.19	12.78	**1.83 (1.37; 2.43)**	**1.43 (1.05; 1.93)**			1.11 (0.80; 1.54)
Father’s educational status								
No education	85.35	4.44	10.22	**1.88 (1.43; 2.47)**		**2.10 (1.46; 2.01)**		**1.71 (1.16; 2.49)**
Primary	87.61	3.63	8.76	**1.55 (1.04; 2.32)**		1.44 (0.92; 2.27)		1.24 (0.77; 1.99)
Secondary	89.98	3.48	6.54	1.22 (0.83; 1.77)		1.19 (0.79; 1.80)		1.18 (0.77; 1.79 )
Above secondary	91.62	2.83	5.55	1		1		**1**
Mother’s educational status								
No education	87.67	4.07	8.26	1.19 (0.89; 1.59)		**0.61 (0.42; 0.87)**		**0.60 (0.41; 0.88)**
Primary	86.18	5.53	8.29	1.34 (0.93; 1.94)		1.03 (0.68; 1.57)		1.05 (0.68; 1.62)
Secondary	89.02	2.64	8.33	1.06 (0.72; 1.54)		1.05 (.70; 1.58)		1.08 (1.08; 0.72)
Above secondary	89.54	2.65	7.81	1		1		**1**
Family size								
One to three	90.19	2.87	6.94	1		1		
Four to seven	89.15	3.62	7.24	1.11 (0.78; 1.57)		1.05 (0.73; 1.52)		1.02 (0.71; 1.48)
Eight and above	83.15	4.75	12.10	**1.86 (1.27; 2.73)**		**1.63 (1.09; 2.43)**		1.35 (0.89; 2.05)
Household wealth								
Lowest	85.13	4.49	10.38	**1.43 (1.07; 1.91)**		1.13 (0.82; 1.54)		0.94 (0.68; 1.32)
Middle	90.50	3.01	6.49	0.85 (0.63; 1.17)		0.83 (0.61; 1.14)		0.83 (0.60; 1.14)
Highest	89.08	3.74	7.18	1		1		**1**
Currently living with								
Both parents	87.62	3.82	8.56	1		1		**1**
One parent	88.09	3.02	8.88	0.96 (0.72; 1.28)		1.02 (0.75; 1.39)		1.09 (0.79; 1.49)
Others	90.66	4.29	5.05	0.72 (0.50; 1.03)		0.69 (0.48; 1.01)		0.69 (0 .47; 1.01)
Presence of mental illness in the family								
No	90.23	3.30	6.47	1		1		**1**
Yes	73.37	6.78	19.85	**3.39 (2.64; 4.35)**		**3.13 (2.42; 4.05)**		**2.01 (1.49; 2.71)**
Residence								
Urban	90.65	2.88	6.47	1			1	1
Rural	74.66	8.25	17.08	**3.24 (2.56; 4.09)**			**2.75 (2.13; 3.55)**	**2.16 (1.61; 2.92)**
School type								
Private	90.70	3.29	6.01	1			1	1
Governmental	86.77	3.98	9.25	**1.49 (1.17; 1.90)**			1.0 (0.77; 1.30)	0.98 (0.75; 1.31)
Frequency of being bullied by others								
Not bullied	90.71	3.29	6.00	1			1	
Once per-week	79.14	5.76	15.11	**2.59 (1.70; 3.98)**			**2.12 (1.37; 3.29)**	1.45 (0.90; 2.33)
Twice or more per week	76.01	5.73	18.26	**3.14 (2.45; 4.03)**			**2.69 (2.08; 3.47)**	**1.79 (1.32; 2.43)**

Bold numbers indicate statistically significant (p-value < 0.05) association.

## Discussion

The present study aimed to investigate the prevalence of internalizing and externalizing problems among in-school adolescents and identify factors associated with these issues. Our results indicate that 24.17% of in-school adolescents experienced internalizing problems, while 11.93% experienced externalizing problems. Furthermore, we found that a high level of internalizing problem score was more likely among those who were female, rural residents, alcohol users, attending public schools, being bullied at school, and being in the lowest wealth index. Similarly, the likelihood of a high-level externalizing problem score was high among alcohol users, adolescents whose fathers are not educated, being rural, and being bullied at school.

The current finding indicates a relatively lower level of mental health problems among adolescents compared to findings from other sub-Saharan countries. Studies conducted in Ghana^74^ and Tanzania^75^ with similar measurements and settings reported higher prevalence than this study, respectively. The variation might originate from the screening tools, population characteristics, and cultural differences across the study settings. Furthermore, adolescents in the current study may lack awareness of their mental health problems or fear the stigma of disclosing their mental health status. However, this finding was higher than the international report^14,15^, but agrees with the national level report that places the prevalence of the problem between 15–25%^17^.

Regardless of the diversity of the measurement tools, sample size, and analysis techniques, all these studies show convergent results that indicate mental health problems are common among adolescents. This finding confirmed that adolescent mental health problems are global issues, and occur at different social levels^76^, and adolescence is a typical age of onset of the problems^77,78^. Therefore, understanding adolescent development across different cultures^79,80^ and addressing mental health problems during the teenage period can have positive lifelong impacts^81^. This finding also indicates that mental health problems are a concern among in-school adolescents in Ethiopia.

The prevalence of adolescent mental health problems was higher in rural residences compared to urban ones. The result is similar to findings from India^82^, China^83^, and West Java^84^. This higher prevalence in rural areas explains the residence as one of the socioeconomic factors associated with adolescents' mental health problems^16^. Such situations occur because family and peer relationships and other environmental factors may also be based on residence. Urban and semi-urban or rural areas have different family, peers, and community environment characteristics. This difference is likely related to the characteristics of living in rural areas where there is a relatively higher burden of poverty and poor education opportunities^83^. Additionally, living in rural areas may expose adolescents to a range of psychological adversities, including increased exposure to crime and violence, alcohol drinking, khat chewing, parental alcoholism, and more negative parental parenting style.

This study also showed that studying in public schools was significantly associated with a higher internalizing problems score among adolescents. Inline the study conducted among school-going adolescent girls in Gujarat, India, adolescents in public schools have higher mental health problems than private school adolescents^85^. The plausible explanation might be a higher chance of socioeconomic hardship among public school students from lower‑income families. Various studies reported that adolescents from low socioeconomic families are at a higher risk of experiencing mental health problems than affluent families^86–88^.

These findings show that adolescents bullied at school are more likely to suffer from mental health issues than those who are not. This finding is consistent with earlier studies that showed a direct connection between bullying at school and adolescent mental health problems. Bullying can cause a range of negative emotions, including fear, sadness, anger, and shame^89^. Bullying can impair physical and mental health, lower academic performance, lower school participation, increase the risk of substance use and suicide, and cause other outcomes, according to a report by StopBullying.gov^90^. Teenagers who have experienced mental health issues are more likely to be bullied. The most significant of these are bullying's psychopathological effects^91^. Therefore, it is essential to recognize the signs of bullying and take appropriate action to prevent it. If you or someone you know is being bullied or has been bullied in the past, it is essential to seek help from a trusted adult or mental health professional. Many online resources can provide information on recognizing the signs of bullying and how to take action against it.

The findings revealed that internalizing and externalizing problems were associated with alcohol use. This finding is supported by a WHO report that reveals the use of alcohol during adolescence is associated with behavioral and emotional problems^92,93^. Studies also showed that the likelihood of developing mental health problems among alcohol users adolescents increases when they engage in risky sexual behavior or episodes of violence which in turn leads to mental health problems^93,94^. Evidence also showed that having a history of alcohol use is associated with mental health disorders such as anxiety and depression during adolescence and later in life. Substance abuse can lead to difficulties with schoolwork, relationship problems, and loss of interest in normal healthy activities, impaired memory, and thinking ability which increases the risk of mental health problems^93,95^. It is important to note that the brain goes through significant changes during adolescence and is particularly vulnerable to the effects of alcohol. Drinking alcohol as a teenager can increase the risk of harm to the developing brain and lead to trouble with alcohol later in life.

Our findings indicated that the father’s educational level was associated with externalizing problems. Adolescents whose fathers were uneducated had more externalizing problems than educated fathers. These associations were in line with previous studies of familiar factors associated with adolescent mental health problems^87,96^. The association might be due to the different roles of parents in nurturing or caring for their children. Another reason may be that educated parents experience stressful life events less than uneducated parents or are better equipped to handle stressful life situations. Additionally, parents with higher education described lower perceived stress and more fabulous control experiences in everyday life^97–99^.

Adolescents from larger family sizes had a higher level of externalizing mental problems than their counterparts. Findings of previous studies also reported similar results^100,101^. This may be well explained by heavier economic or social burdens placed on large families. Studies revealed that financial constraints and economic hardships increase behavioral problems of individual and their families^102^ that are expected from larger family members. In addition to this adolescents from larger family sizes may tend to get less support, attention, individual care, and supervision from their family as they are increased in number which in turn triggers mental health problems^103,104^.

### Strengths and limitations of the study

Our large sample size of 3326 participants enhances the statistical power and the generalizability of our findings. Our study encompasses a wide demographic, including students from both urban and rural backgrounds, and public and private schools. This varied sample enhances the representativeness of our study, thereby boosting the applicability of our findings to a broader population. We employed validated, guided self-administered questionnaires for data collection. This method not only ensures the reliability and validity of our data but also minimizes bias and enhances the precision of the responses.

Even though it provides novel information regarding in-school adolescents, there are significant limitations to be considered in interpreting the findings of this study. In this study, a self-report version of SDQ was used to collect the data even though data from multiple informants are often more reliable than data from single informants. The study adopted a cross-sectional descriptive study design covering twenty-three schools in the Harari regional state; therefore, the findings of this study may not be generalizable to all school-going adolescents in eastern Ethiopia. Adolescents were asked about problems they encountered in the past 6 months that may lead to a chance of recall bias. However, we attempted to minimize this bias by using validated questionnaires and by ensuring anonymity to encourage honest responses. We also recognize that participants may have responded in a way that they believe is socially acceptable rather than reflecting their true feelings or behaviors. We addressed this by emphasizing to participants that there were no right or wrong answers and that their responses would remain confidential.

The study did not include out‑of‑school adolescents. These disparities could have influenced our results, and we suggest that future research should aim to include a more evenly distributed sample. Finally, the data collected during the COVID-19 pandemic might influence the perceptions of students and can affect the results. While our study does not directly investigate the impact of the COVID-19 pandemic on adolescents’ mental health, we recognize that the pandemic and its associated disruptions may have influenced the mental health landscape for this demographic. The abrupt shift to online learning, isolation from peers, and the general climate of fear and uncertainty could potentially intensify the internalizing and externalizing mental health problems we explore in this study.

## Conclusion

The study found that a significant proportion of in-school adolescents experienced either internalizing or externalizing mental problems. Females, rural residents, alcohol users, those bullied, and students attending public schools had high internalizing problem scores. Externalizing problem scores were high among alcohol users, adolescents whose fathers are uneducated, and rural residents.

To address the specific needs of the population, schools and communities should prioritize mental health awareness and support programs for adolescents. Females with high internalizing problem scores should be encouraged to seek professional counseling services and develop effective coping mechanisms. They should also be provided with access to mental health resources such as online support groups, self-help books, and educational materials to learn more about their condition and develop strategies to manage their symptoms. Stress management programs such as yoga, meditation, and mindfulness training can also be recommended to help females manage their stress levels and improve their emotional well-being.

It is important to address the issue of alcohol use among adolescents, which was found to be associated with both internalizing and externalizing problems. Future research should continue to explore gender differences in adolescent mental health to inform the development of more effective, targeted interventions.

The study’s findings have several implications for policy and practice. Policymakers should consider investing in mental health services and resources for adolescents, particularly in rural areas. Schools should also consider implementing mental health screening programs to identify students who may be at risk of developing mental health problems.

## Data Availability

The datasets analyzed for this manuscript are available from the corresponding author upon a reasonable request.

## Abbreviations

ADHD: Attention deficit hyperactivity disorder
AOR: Adjusted odds ratio
CD: Conduct disorders
SDQ: Strength and difficulty questionnaire

## References

[1] Achenbach TM, Dumenci L, Rescorla LA (2001). Ratings of Relations Between DSM-IV Diagnostic Categories and Items of the CBCL/6-18, TRF, and YSR.

[2] Keil V, Price JM (2006). Externalizing behavior disorders in child welfare settings: Definition, prevalence, and implications for assessment and treatment. Child Youth Serv. Rev..

[3] American Psychiatric Association (2013). American Psychiatric Association: Diagnostic and Statistical Manual of Mental Disorders.

[4] World Health Organization (2017). Mental Health Status of Adolescents in South-East Asia: Evidence for Action.

[5] World Health Organization (2018). Adolescent Mental Health.

[6] Chaney M, Jani S, Shekunov J, Choice T (2017). Adolescent Mental Health Disorders.

[7] Keeley B (2021). The State of the World's Children 2021: On My Mind—Promoting, Protecting and Caring for Children's Mental Health.

[8] Nayak RB, Patil S, Patil N, Chate SS, Koparde VA (2015). Psychiatric morbidity among inmates of center for destitutes: A cross-sectional study. J. Sci. Soc..

[9] WHO (2017). Global Accelerated Action for the Health of Adolescents (AA-HA!): Guidance to Support Country Implementation: Summary.

[10] Mack A (2012). Cumulative prevalence of psychiatric disorders by young adulthood: A prospective cohort analysis from the Great Smoky Mountains study. Year Book Psychiatry Appl. Ment. Health.

[11] Costello EJ, Copeland W, Angold A (2011). Trends in psychopathology across the adolescent years: What changes when children become adolescents, and when adolescents become adults?. J. Child Psychol. Psychiatry.

[12] Fergusson DM, Boden JM, Horwood LJ (2007). Recurrence of major depression in adolescence and early adulthood, and later mental health, educational and economic outcomes. Br. J. Psychiatry.

[13] Eyre O, Thapar A (2014). Common adolescent mental disorders: Transition to adulthood. Lancet.

[14] UNDESA. United Nations Department of Economic and Social Affairs (UNDESA) World Population Prospects 2019 (UNDESA, 2021). https://population.un.org/wpp/DataQuery/. Accessed 31 May 2021.

[15] IHME. GBD Results Tool (2021). http://ghdx.healthdata.org/gbd-results-tool. Accessed 9 Sept 2021.

[16] Cortina MA, Sodha A, Fazel M, Ramchandani PG (2012). Prevalence of child mental health problems in sub-Saharan Africa: A systematic review. Arch. Pediatr. Adolesc. Med..

[17] Ethiopian Federal Ministry of Health (2016). National Adolescent and Youth Health Strategy (2016–2020).

[18] Patton GC, Sawyer SM, Santelli JS, Ross DA, Afifi R, Allen NB (2016). Our future: A Lancet commission on adolescent health and wellbeing. Lancet.

[19] World Health Organization (2017). Global Accelerated Action for the Health of Adolescents (AA-HA!): Guidance to Support Country Implementation.

[20] The World Bank. Mental health 2018. https://www.worldbank.org/en/topic/mental-health (2018).

[21] World Health Organization (2016). mhGAP Intervention Guide for Mental, Neurological and Substance Use Disorders in Non-specialized Health Settings. Version 2.0.

[22] World Health Organization. Adolescent mental health 2019. https://www.who.int/mental_health/maternal-child/adolescent/en/ (2019).

[23] Patel V, Saxena S, Lund C, Thornicroft G, Baingana F, Bolton P (2018). The Lancet Commission on global mental health and sustainable development. Lancet.

[24] World Health Organization. Adolescent and young adult health. https://www.who.int/news-room/fact-sheets/detail/adolescents-health-risks-and-solutions (2021).

[25] Mental Health Foundation (2018). What New Statistics Show About Children's Mental Health.

[26] Whiteford HA, Degenhardt L, Rehm J, Baxter AJ, Ferrari AJ, Erskine HE (2013). Global burden of disease attributable to mental and substance use disorders: Findings from the Global Burden of Disease Study 2010. Lancet.

[27] Kwon E, Kim B, Lee H, Park S (2018). Heterogeneous trajectories of depressive symptoms in late middle age: Critical period, accumulation, and social mobility life course perspectives. J. Aging Health.

[28] Wüstner A, Otto C, Schlack R, Hölling H, Klasen F, Ravens-Sieberer U (2019). Risk and protective factors for the development of ADHD symptoms in children and adolescents: Results of the longitudinal BELLA study. PLoS One.

[29] Senol V, Unalan D, Akca RP, Basturk M (2018). Prevalence of attention-deficit/hyperactivity and other disruptive behaviour disorder symptoms among primary school-age children in Kayseri, Turkey. J. Int. Med. Res..

[30] Liakoni E, Gartwyl F, Ricklin M, Exadaktylos AK, Krahenbuhl S (2018). Psychoactive substances and violent offences: A retrospective analysis of presentations to an urban emergency department in Switzerland. PLoS One.

[31] Wang GS, Hoyte C (2018). Common substances of abuse. Pediatr. Rev..

[32] Silke B (2018). Epidemiology of suicide and the psychiatric perspective. Int. J. Environ. Res. Public Health.

[33] Beardslee WR, Brent DA, Weersing VR, Clarke GN, Porta G, Hollon SD (2013). Prevention of depression in at-risk adolescents: Longer-term effects. JAMA Psychiatry.

[34] Lamis DA, Dvorak RD (2014). Mindfulness, nonattachment, and suicide rumination in college students: The mediating role of depressive symptoms. Mindfulness.

[35] Kennedy B, Chen R, Valdimarsdottir U, Montgomery S, Fang F, Fall K (2018). Childhood bereavement and lower stress resilience in late adolescence. J. Adolesc. Health.

[36] Stikkelbroek Y, Bodden DH, Reitz E, Vollebergh WA, van Baar A (2016). Mental health of adolescents before and after the death of a parent or sibling. Eur. Child Adolesc. Psychiatry.

[37] Siegel J, Han W-J (2018). Family exposure to potentially traumatic events and Chinese children’s psychological adjustment: A transgenerational study. J. Child Family Stud..

[38] Ford R, King T, Priest N, Kavanagh A (2017). Bullying and mental health and suicidal behaviour among 14-to 15-year-olds in a representative sample of Australian children. Aust. N. Z. J. Psychiatry.

[39] Le HTH, Tran N, Campbell MA, Gatton ML, Nguyen HT, Dunne MP (2019). Mental health problems both precede and follow bullying among adolescents and the effects differ by gender: A cross-lagged panel analysis of school-based longitudinal data in Vietnam. Int. J. Ment. Health Syst..

[40] Moksnes UK, Reidunsdatter RJ (2019). Self-esteem and mental health in adolescents—Level and stability during a school year. Norsk Epidemiologi.

[41] Ngo H, VanderLaan DP, Aitken M (2020). Self-esteem, symptom severity, and treatment response in adolescents with internalizing problems. J. Affect. Disord..

[42] Wang S, Xu H, Zhang S, Yang R, Li D, Sun Y (2020). Linking childhood maltreatment and psychological symptoms: The role of social support, coping styles, and self-esteem in adolescents. J. Interpers. Violence.

[43] World Health Organization. COVID-19 pandemic triggers 25% increase in prevalence of anxiety and depression worldwide. https://www.who.int/news/item/02-03-2022-covid-19-pandemic-triggers-25-increase-in-prevalence-of-anxiety-and-depression-worldwide (2023). PMC9998057 35414629

[44] Patrick SW, Henkhaus LE, Zickafoose JS, Lovell K, Halvorson A, Loch S (2020). Well-being of parents and children during the COVID-19 pandemic: A national survey. Pediatrics.

[45] Gassman-Pines A, Ananat EO, Fitz-Henley J (2020). COVID-19 and parent-child psychological well-being. Pediatrics.

[46] Ezpeleta L, Navarro JB, de la Osa N, Trepat E, Penelo E (2020). Life conditions during COVID-19 lockdown and mental health in Spanish adolescents. Int. J. Environ. Res. Public Health.

[47] Orgilés M, Morales A, Delvecchio E, Mazzeschi C, Espada JP (2020). Immediate psychological effects of the COVID-19 quarantine in youth from Italy and Spain. Front. Psychol..

[48] Jiao WY, Wang LN, Liu J, Fang SF, Jiao FY, Pettoello-Mantovani M (2020). Behavioral and emotional disorders in children during the COVID-19 epidemic. J. Pediatr..

[49] Xie X, Xue Q, Zhou Y, Zhu K, Liu Q, Zhang J (2020). Mental health status among children in home confinement during the coronavirus disease 2019 outbreak in Hubei Province, China. JAMA Pediatr..

[50] Yeasmin S, Banik R, Hossain S, Hossain MN, Mahumud R, Salma N (2020). Impact of COVID-19 pandemic on the mental health of children in Bangladesh: A cross-sectional study. Child. Youth Serv. Rev..

[51] Saurabh K, Ranjan S (2020). Compliance and psychological impact of quarantine in children and adolescents due to Covid-19 pandemic. Indian J. Pediatr..

[52] Garcia de Avila MA, Hamamoto Filho PT, Jacob FLS, Alcantara LRS, Berghammer M, Jenholt Nolbris M (2020). Children’s anxiety and factors related to the COVID-19 pandemic: An exploratory study using the children’s anxiety questionnaire and the numerical rating scale. Int. J. Environ. Res. Public Health.

[53] Langmeyer A, Guglhör-Rudan A, Naab T, Urlen M, Winklhofer U (2020). Kindsein in Zeiten von Corona. Erste Ergebnisse zum veränderten Alltag und zum Wohlbefinden von Kindern.

[54] Fekadu D, Alem A, Hägglöf B (2006). The prevalence of mental health problems in Ethiopian child laborers. J. Child Psychol. Psychiatry.

[55] Ashenafi Y, Kebede D, Desta M, Alem A (2001). Prevalence of mental and behavioral disorders in children in Ethiopia. East Afr. Med. J..

[56] Desta, M., Hägglöf, B., Kebede, D. & Alem, A. Psychiatric disorders in urban children in Ethiopia: A population based cross sectional survey. *Soc. Psychiatry Psychiatr. Epidemiol*. (2008).

[57] Ministry of Health. National mental health strategy, 2020–2025 (2013–2017 EFY) Ministry of Health (Ministry of Health—Ethiopia, 2020-01-01) (2020).

[58] Hunduma G, Dessie Y, Geda B, Yadeta TA, Deyessa N (2022). Internalizing and externalizing mental health problems affect in-school adolescent’s health-related quality of life in eastern Ethiopia: A cross-sectional study. PLoS One.

[59] FDROE, MOH (2016). National Adolescent and Youth Health Strategy (2016–2020).

[60] Central Statistical Authority (CSA). The 2007 Population and Housing Census of Ethiopia: “Statistical Report for Harari Region” for the census; Population and housing census of 2007 (Central Statistical Agency-Ministry of Finance and Economic Development, 2007).

[61] Harari BoFED. Harari Bureau of Finance and Economic Development, Baseline Data of Harari region (Harar, 2010).

[62] Ahmed, W. M. *History of Harar and the Hararis* (Harari People Regional State Culture, Heritage and Tourism Bureau, 2008).

[63] Harar Urban HDSS INDEPTH Core Dataset 2012–2016 (Release 2018). Version: v1. INDEPTH Network. Dataset. 10.7796/INDEPTH.ET042.CMD2016.v1 [Internet] (2018).

[64] Harari Education Office (2018). Harari Educational Office Quick Indicator Reference of the Year 2017/18 Academic Year.

[65] FDROE MOH,  (2016). National Adolescent and Youth Health Strategy (2016–2020) Federal Democratic Republic of Ethiopia Ministry of Health.

[66] Goodman R, Ford T, Simmons H, Gatward R, Meltzer H (2000). Using the Strengths and Difficulties Questionnaire (SDQ) to screen for child psychiatric disorders in a community sample. Br. J. Psychiatry.

[67] Meltzer H, Gatward R, Goodman R, Ford T (2003). Mental health of children and adolescents in Great Britain. Int. Rev. Psychiatry.

[68] Goodman R, Meltzer H, Bailey V (1998). The strengths and difficulties questionnaire: A pilot study on the validity of the self-report version. Eur. Child Adolesc. Psychiatry.

[69] Boyer NR, Miller S, Connolly P, McIntosh E (2016). Paving the way for the use of the SDQ in economic evaluations of school-based population health interventions: An empirical analysis of the external validity of SDQ mapping algorithms to the CHU9D in an educational setting. Qual. Life Res..

[70] Information for researchers and professionals about the Strengths and Difficulties Questionnaire. http://www.sdqinfo.com/. Accessed 18 Sept 2021. Archived by WebCite at http://www.webcitation.org/6YH56saPy 10.1111/j.1475-3588.2007.00443_2.x 32811128

[71] Goodman A, Goodman R (2009). Strengths and difficulties questionnaire as a dimensional measure of child mental health. J. Am. Acad. Child Adolesc. Psychiatry.

[72] Goodman A, Lamping DL, Ploubidis GB (2010). When to use broader internalising and externalising subscales instead of the hypothesised five subscales on the Strengths and Difficulties Questionnaire (SDQ): Data from British parents, teachers and children. J. Abnorm. Child Psychol..

[73] Central Statistical Agency (CSA) [Ethiopia] and ICF. Ethiopia Demographic and Health Survey 2016 (CSA and ICF, 2016).

[74] Addy ND, Agbozo F, Runge-Ranzinger S, Grys P (2021). Mental health difficulties, coping mechanisms and support systems among school-going adolescents in Ghana: A mixed-methods study. PLoS One.

[75] Nkuba M, Hermenau K, Goessmann K, Hecker T (2018). Mental health problems and their association to violence and maltreatment in a nationally representative sample of Tanzanian secondary school students. Soc. Psychiatry Psychiatr. Epidemiol..

[76] United Nations International Children’s Emergency Fund. Communication for Development (C4D): Global Progress and Country Level Highlights Across Programme Areas. https://www.unicef.org/publications/index_102938.html (2018).

[77] Matos A, Salvador M, Costa J, Pinheiro M, Arnarson E, Craighead W (2017). The relationship between internalizing and externalizing problems in adolescence: Does gender make a difference?. Can. Int. J. Soc. Sci. Educ..

[78] Fisher JR, de Mello MC (2011). Using the World Health Organization's 4S-framework to strengthen national strategies, policies and services to address mental health problems in adolescents in resource-constrained settings. Int. J. Ment. Health Syst..

[79] Tamnes CK, Herting MM, Goddings A-L, Meuwese R, Blakemore S-J, Dahl RE (2017). Development of the cerebral cortex across adolescence: A multisample study of inter-related longitudinal changes in cortical volume, surface area, and thickness. J. Neurosci..

[80] Blakemore S-J (2019). Adolescence and mental health. Lancet.

[81] Carvajal-Velez L, Requejo JH, Ahs JW, Idele P, Adewuya A, Cappa C (2023). Increasing data and understanding of adolescent mental health worldwide: UNICEF’s measurement of mental health among adolescents at the population level initiative. J. Adolesc. Health.

[82] Pandia V, Noviandhari A, Amelia I, Hidayat GH, Fadlyana E, Dhamayanti M (2021). Association of mental health problems and socio-demographic factors among adolescents in Indonesia. Glob. Pediatr. Health.

[83] Wang JN, Liu L, Wang L (2014). Prevalence and associated factors of emotional and behavioural problems in Chinese school adolescents: A cross-sectional survey. Child Care Health Dev..

[84] Nkuba, M. Child maltreatment, mental health problems and prevention of violence among secondary school students in Tanzania (2017).

[85] Mangal A, Thakur A, Nimavat KA, Dabar D, Yadav SB (2020). Screening for common mental health problems and their determinants among school-going adolescent girls in Gujarat, India. J. Family Med. Primary Care.

[86] Reiss F (2013). Socioeconomic inequalities and mental health problems in children and adolescents: A systematic review. Soc. Sci. Med..

[87] Reiss F, Meyrose A-K, Otto C, Lampert T, Klasen F, Ravens-Sieberer U (2019). Socioeconomic status, stressful life situations and mental health problems in children and adolescents: Results of the German BELLA cohort-study. PLoS One.

[88] Heshmat R, Qorbani M, Ghoreshi B, Djalalinia S, Tabatabaie OR, Safiri S (2016). Association of socioeconomic status with psychiatric problems and violent behaviours in a nationally representative sample of Iranian children and adolescents: The CASPIAN-IV study. BMJ Open.

[89] Arseneault L (2017). The long-term impact of bullying victimization on mental health. World Psychiatry.

[90] Wolke D, Lereya ST (2015). Long-term effects of bullying. Arch. Dis. Child..

[91] Källmén H, Hallgren M (2021). Bullying at school and mental health problems among adolescents: A repeated cross-sectional study. Child Adolesc. Psychiatry Ment. Health.

[92] Auerbach RP, Alonso J, Axinn WG, Cuijpers P, Ebert DD, Green JG (2016). Mental disorders among college students in the World Health Organization world mental health surveys. Psychol. Med..

[93] World Health Organization. Adolescent and Young Adult Health. https://www.who.int/news-room/fact-sheets/detail/adolescents-health-risks-and-solutions (2023).

[94] Miller C (2020). Mental Health Disorders and Teen Substance Use.

[95] Richert T, Anderberg M, Dahlberg M (2020). Mental health problems among young people in substance abuse treatment in Sweden. Subst. Abuse Treat. Prev. Policy.

[96] Luo Y, Cui Z, Zou P, Wang K, Lin Z, He J (2020). Mental health problems and associated factors in chinese high school students in Henan Province: A cross-sectional study. Int. J. Environ. Res. Public Health.

[97] Meyrose A-K, Klasen F, Otto C, Gniewosz G, Lampert T, Ravens-Sieberer U (2018). Benefits of maternal education for mental health trajectories across childhood and adolescence. Soc. Sci. Med..

[98] Rajmil L, Herdman M, Ravens-Sieberer U, Erhart M, Alonso J (2014). Socioeconomic inequalities in mental health and health-related quality of life (HRQOL) in children and adolescents from 11 European countries. Int. J. Public Health.

[99] Mandemakers JJ, Monden CW (2010). Does education buffer the impact of disability on psychological distress?. Soc. Sci. Med..

[100] Kylmänen P, Hakko H, Räsänen P, Riala K (2010). Is family size related to adolescence mental hospitalization?. Psychiatry Res..

[101] Hatcher S, Coupe N, Wikiriwhi K, Durie M, Pillai A (2016). Te Ira Tangata: A Zelen randomised controlled trial of a culturally informed treatment compared to treatment as usual in Māori who present to hospital after self-harm. Soc. Psychiatry Psychiatr. Epidemiol..

[102] Knapp M, Wong G (2020). Economics and mental health: The current scenario. World Psychiatry.

[103] Daley DC (2013). Family and social aspects of substance use disorders and treatment. J. Food Drug Anal..

[104] Halonen J, Hakko H, Riala K, Riipinen P (2022). Familial risk factors in relation to recurrent depression among former adolescent psychiatric inpatients. Child Psychiatry Hum. Dev..

